# Impact of design, size, and a combined routine on the plaque removal efficacy of manual, hybrid (powered-mode) and interdental toothbrushes: a robot-assisted in vitro study

**DOI:** 10.1186/s12903-026-09584-w

**Published:** 2026-08-18

**Authors:** Daniel Bacqueville, Mélanie Souyris, Tomas Lang, Alberto Ogalla, Aurélien Simonini, Peter Gängler, Nathalie Castex-Rizzi

**Affiliations:** 1 Pierre Fabre Dermocosmetics and Personal Care, Research & Development, Scientific Evaluation / Pharmacology Department, Toulouse, France; 2 ORMED Institute for Oral Medicine, University of Witten/Herdecke UG, Witten, Germany; 3 Pierre Fabre Dermocosmetics and Personal Care, Pierre Fabre Oral Care, Medical and Patient/Consumer Relationship Department, Lavaur, France; 4 Pierre Fabre Dermocosmetics and Personal Care, Pierre Fabre Oral Care, Dental accessories and medical devices development, Lavaur, France

**Keywords:** Simulated dental plaque, Manual and hybrid toothbrushes, Interdental toothbrushes, Robot testing, Hygienist, Oral hygiene

## Abstract

**Background:**

Regular and effective toothbrushing with interdental cleaning is key to maintaining oral health. Using an in vitro testing system, we compared the plaque removal efficacy of manual toothbrushes with different bristle designs, different-sized interdental brushes (IDBs), and a hybrid brush used in powered mode alone or in a combined routine with IDBs.

**Methods:**

Manual (*n* = 4) and powered (hybrid model; sonic mode) toothbrushing was simulated using a clinically tested robot programme (rotating movement, 2 min, 3.5 N force). IDBs (*n* = 8) were evaluated using a standardized hygienist protocol. Tests were carried out on a typodont tooth model and cleaning efficacy was evaluated using computer-assisted optical automatic plaque planimetry. Data were analysed using descriptive statistics, the Kolmogorov-Smirnov test, two-sample *t* test and Mann-Whitney *U* test.

**Results:**

Manual toothbrushes with longer, soft and conical-shaped bristles showed the highest levels of overall in vitro simulated plaque removal: up to 72.6% with improved cleaning efficacy for interdental, gum-line, and root fields under test conditions. IDBs ≥ 1.3 mm were the most effective for removing simulated dental plaque at the poorly accessible mesial and distal sites of the typodont model (up to 77.1%). The combined hybrid powered-mode toothbrushing and hygienist IDB protocol resulted in a significant 15.1% increase in simulated plaque removal (81.5% overall), with the greatest improvement in interdental surface and root fields (values up to 88%).

**Conclusions:**

These in vitro data provided insights into design characteristics that could potentially improve the cleaning efficacy of manual brushes and highlighted the importance of using appropriately sized IDBs. They also showed that the robot testing system could be used to test hybrid brushes in powered mode, as well as alongside standardized hygienist IDB protocol to evaluate the potential efficacy of combined cleaning routines, which may be useful for providing guidance during the early phases of development of new devices and product ranges.

**Supplementary Information:**

The online version contains supplementary material available at https://doi.org/10.1186/s12903-026-09584-w.

## Background

Dental plaque is the principal cause of periodontal disease and the formation of dental caries [1]. Encouraging individuals to maintain good oral hygiene and ensure they regularly eliminate the buildup of supragingival dental biofilm is the first step in preserving dental and periodontal health [1–3]. Mechanical plaque removal through regular toothbrushing, with either manual or powered toothbrushes, is the principal means of disease prevention [3, 4]. Although these conventional brushes allow adequate cleaning of the buccal, lingual, and occlusal tooth surfaces, combining toothbrushing with the use of interdental cleaning devices has been shown to further improve oral hygiene by allowing better access to hard-to-reach proximal areas and interdental spaces [1, 3, 5–8]. 

The efficacy of toothbrushing for plaque removal can also be improved through oral hygiene education, which provides patients with instructions on optimal brushing movements, duration and frequency; however, patient factors, such as motivation and manual dexterity, may still influence outcomes [9–13]. Many innovative manual and powered toothbrushes aim to compensate for inadequate brushing and poor technique with designs that help users reach areas that are difficult to access and with improved ergonomics to motivate individuals to adopt oral hygiene-friendly behaviour [14]. Manufacturers have also developed brushes with different bristle characteristics (e.g., variations in bristle tuft number, pattern, hardness, length and diameter), various head designs and sizes to improve plaque removal in proximal areas, and assorted handles for better grip [14–17]. In addition, powered toothbrushes are also available with different movement patterns (side-to-side, counter-oscillation, rotation-oscillation, and multidimensional motions), vibration speeds (standard, sonic and ultra-sonic), and modes of action (e.g., hybrid brushes with manual, powered or combined manual and sonic modes) that allow users to tailor their brushing routine or technique according to their individual needs [14, 18–21]. Finally, interdental brushes (IDBs) have emerged as an easy-to-use and effective method of interdental cleaning, with a range of different brush shapes and sizes available to optimize plaque removal and account for variations in anatomical characteristics [1, 8, 22, 23]. 

The efficacy of these oral devices is often measured in vivo. However, clinical studies are time-consuming and expensive to conduct. In addition, the results obtained are often difficult to attribute to design innovations because of differences in brushing techniques between trial participants and hard to compare because of great variability between study protocols and a lack of standardization across study designs [1, 4, 19]. In vitro assessments of tooth cleaning efficacy provide a cost-effective and easy-to-standardize method for testing the potential benefits of device designs in the early stages of product development, prior to wider clinical testing. Several sophisticated in vitro methods have been developed for this purpose; however, the system described by Lang et al. (2014), which couples the use of a standardized robotic toothbrushing simulation programme on a typodont tooth model with clinical planimetric index assessments of toothbrush cleaning efficacy, was the first such testing method considered to be clinically validated [24]. More recently, this robotic system has been used alongside computer-assisted optical automatic plaque planimetry (APP) to provide reproducible and standardized assessments of the impact of manual toothbrush design parameters on simulated plaque removal efficacy [15]. 

Here, we describe the results of a series of in vitro studies that used this same typodont model-robot simulation–APP testing system to evaluate the in vitro simulated plaque removal efficacy of several commercially available manual toothbrushes with various bristle designs, and, for the first time, of a hybrid toothbrush used in powered mode. In addition, a standardized hygienist-led interdental brushing protocol was developed and used to assess the cleaning efficacy of a range of commercially available IDBs. This interdental brushing protocol was then used in combination with the robot simulation–APP testing system to assess the efficacy of a hybrid powered-mode toothbrush and IDB cleaning regimen.

## Materials and methods

### Aim, design and setting of the study

This series of in vitro studies aimed to (i) use a typodont model–robot simulation–APP testing system to identify the specific features that most impacted the performance of various manual toothbrushes; (ii) use a standardized hygienist-led interdental brushing protocol to objectively assess the efficacy and suitability of a range of commercially available IDBs for use on the typodont model; (iii) use a typodont model-robot simulation–APP testing system to evaluate the in vitro cleaning efficacy of a hybrid toothbrush in the powered mode; and (iv) use the robot simulation–APP system and hygienist IDB protocol in combination to evaluate the in vitro cleaning efficacy of an interdental and hybrid powered-mode toothbrush cleaning regimen.

### Study products

**Fig. 1:**
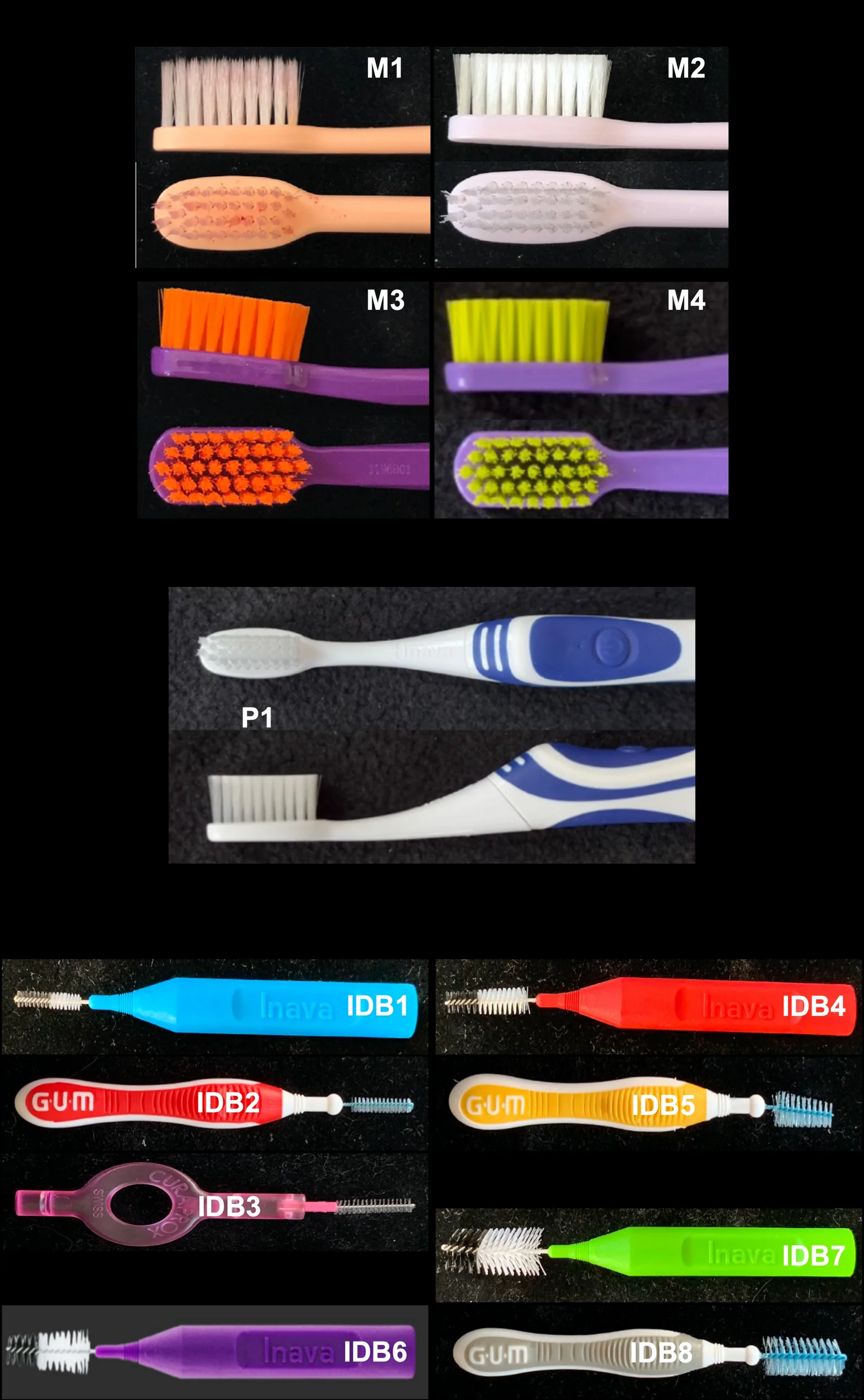
Tested manual toothbrushes (M1 to M4), hybrid powered-mode toothbrush (P1) and interdental brushes (IDB1 to IDB8)

A total of four manual toothbrushes, one hybrid powered-mode toothbrush and eight IDBs, with various designs and features, were evaluated in this study. All toothbrushes were appropriately endrounded and used for the first time during the experimental brushing. The individual characteristics of the study products are summarized in Table 1 and images are provided in Fig. 1.

**Table 1:** Listing and characteristics of the toothbrushes (manual and hybrid powered-mode brushes) and interdental brushes evaluated

Brush type		Reference	Manufacturer	Product name	Design and specific properties
Manual toothbrushes		M1	PFOC^†^	Elgydium Clinic Sensileave	3024 bristles/toothbrush. Hardness: soft. Tuft height: 11 mm. PBT conical bristle Ø: 18/100 mm at the base, 1/100 mm at the top
		M2	PFOC^†^	Inava 15/100	4176 bristles/toothbrush. Hardness: soft. Tuft height: 11 mm. Nylon 6/12 rounded cylindrical bristle Ø: 15/100 mm
		M3	Curaden^‡^	Curaprox 5460	5460 bristles/toothbrush. Hardness: extra-soft. Tuft height: 9 mm. Curen^®^ rounded cylindrical Bristle Ø: 10/100 mm
		M4	Curaden^‡^	Curaprox 1560	1560 bristles/toothbrush. Hardness: soft. Tuft height: 9 mm. Curen^®^ rounded cylindrical bristle Ø: 15/100 mm
Hybrid toothbrush rechargeable (in powered mode)		P1	PFOC^†^	Inava Hybrid	***Design*** Head shape: oval. PBT conical bristle Ø: 18/100 mm at the basis, 1/100 mm at the top ***Mechanical properties*** Neck: thin and flexible. Bristles: soft, high flexibility for a non-traumatic brushing of gums and enamel. Brushing modes: manual, powered (sonic) or combined (manual and sonic). The sonic mode uses vibration technology; the brush head makes side-by-side movements and produces up to 28 000 strokes per minute. Used in powered mode (1x press)
IDBs	Small	IDB1	PFOC^†^	Inava MonoCompact ISO1 Blue (0.8 mm)	2 diameters and 2 colours nylon 6/12 bristles (50% white/50% black). Shape: cylindrical. Brush Ø: 1.9 mm
		IDB2	Sunstar^§^	Gum TRAV-LER ISO1 Red (0.8 mm)	Ultrathin triangular bristles coated with antibacterial agent (chlorhexidine). Shape: cylindrical. Brush Ø: 2.3 mm
		IDB3	Curaden^‡^	Curaprox CPS 08 Pink (0.8 mm)	Ultrathin bristles with umbrella effect. Shape: cylindrical. Brush Ø: 3.2 mm
	Medium	IDB4	PFOC^†^	Inava MonoCompact ISO4 Red (1.5 mm)	2 diameters and 2 colours nylon 6/12 bristles (50% white/50% black). Shape: conical. Brush Ø: 3–4 mm
		IDB5	Sunstar^§^	Gum TRAV-LER ISO4 Yellow (1.3 mm)	Ultrathin triangular bristles coated with antibacterial agent (chlorhexidine). Shape: conical. Brush Ø: 3.1–5.1 mm
	Large	IDB6	PFOC^†^	Inava MonoCompact ISO5 Violet (1.8 mm)	2 diameters and 2 colours nylon 6/12 bristles (50% white/50% black). Shape: conical. Brush Ø: 4–6 mm
	Extra-large	IDB7	PFOC^†^	Inava MonoCompact ISO6 Green (2.2 mm)	2 diameters and 2 colours nylon bristles (50% white/50% black). Shape: conical. Brush Ø: 7–8 mm
		IDB8	Sunstar^§^	Gum TRAV-LER ISO6 Grey (2 mm)	Ultrathin triangular bristles coated with antibacterial agent (chlorhexidine). Shape: cylindric. Brush Ø: 6.9 mm

*Brush Ø* brush diameter, *IDB* interdental brush, *M* manual toothbrush, *P* powered-mode toothbrush, *PBT* polybutylene terephthalate

† Pierre Fabre Oral Care (PFOC), Castres, France; ^‡^ Curaden, Kriens, Switzerland; ^§^ Sunstar, Levallois-Perret, France

### In vitro evaluation system

**Fig. 2:**
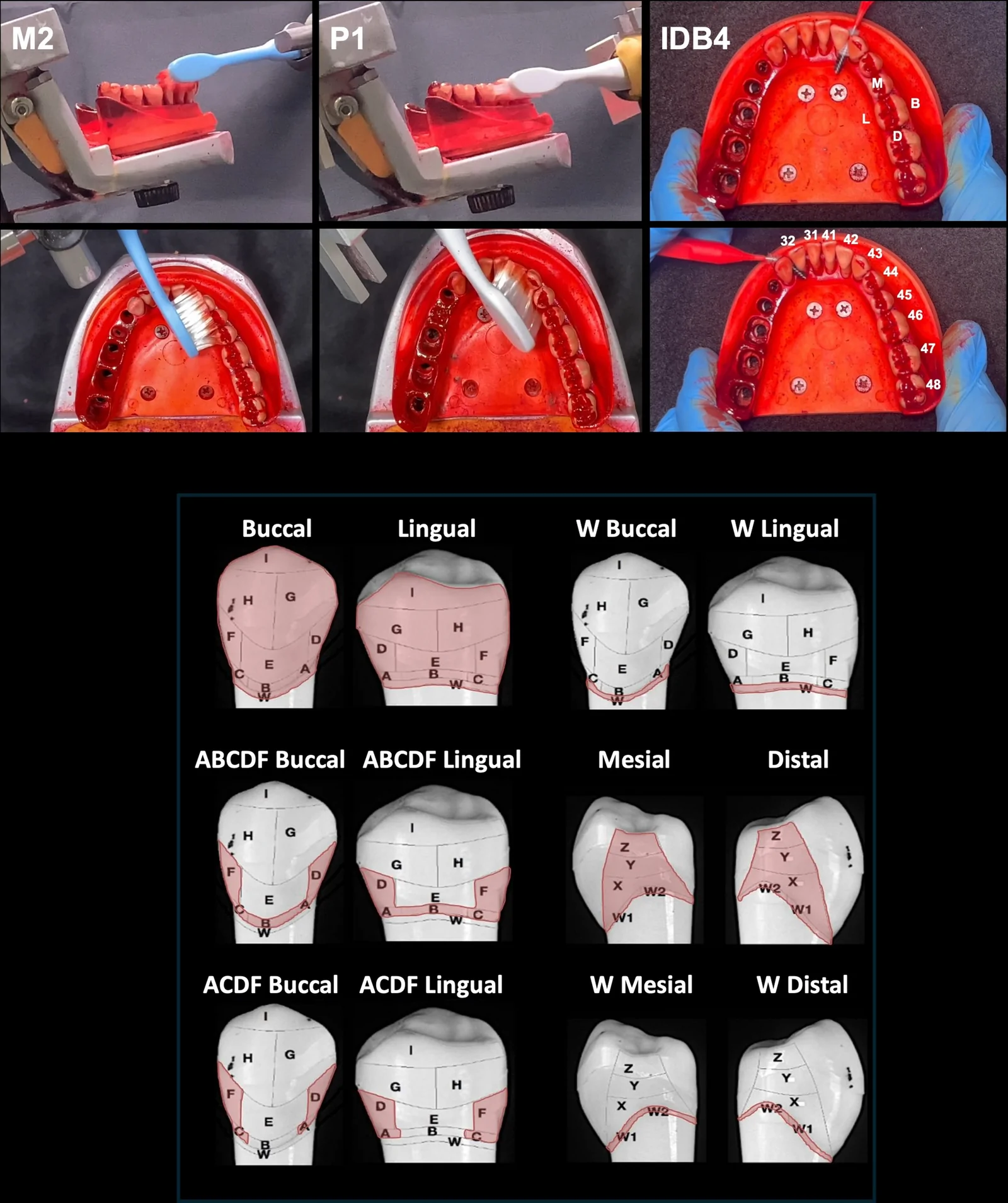
The robotic brushing and computer-assisted optical automatic plaque planimetry evaluation system. **A**. Images of the robot testing system with the manual (M2) and hybrid powered-mode (P1) toothbrushes, and interdental brushing with IDB4 by the hygienist. Letters indicate tooth surfaces: B, buccal; L, lingual; M, mesial; D, distal. Numbers correspond to tooth nomenclature: 32/31/41/42 incisors; 43 canines; 44/45 premolars; and 46/47/48 molars. **B**. The planimetrical fields assessed using the automated plaque planimetry index (a premolar is shown) with the buccal (toward the cheek) and lingual (toward the tongue) surfaces with fields A to I and one root field (W); the between-tooth mesial (anterior) and distal (posterior) surfaces with fields X to Z and two root fields (W1 + W2); and the gum-line ABCDF and at-risk interproximal ACDF fields for toothbrushes and interdental brushes, respectively

The manual toothbrushes, hybrid powered-mode toothbrush, and IDBs were tested on a set of 10 artificial teeth (4 incisors, 1 canine, 2 premolars, 3 molars; kavo basic model teeth, KaVo Dental, Biberach, Germany) covered in simulated plaque (as illustrated in Fig. 2A**)**. Brushing with manual and hybrid powered-mode toothbrushes was performed using a 6-axis robot toothbrushing system (FS002N; Kawasaki Robotics, Akashi, Hyogo, Japan). This robotic, simulated, plaque-brushing system was calibrated according to the clinically validated brushing programme described previously by Lang et al. (2014) [24], using standardized parameters for brushing force and time, movement and angulation. Brushing with the IDB was performed by a hygienist, using a defined set of movements and a specialized template to standardize brushing angles.

The cleaning efficacy of the toothbrushes and IDBs was then evaluated using computer-assisted optical APP to assess the percentage of simulated plaque removed from the artificial teeth during robotic brushing programmes. As described previously [15], APP allows plaque removal to be assessed across a range of up to 30 planimetric fields including buccal, lingual and interdental surfaces and at-risk areas at the root and close to the gum line. A summary of the APP detection fields assessed is provided in Fig. 2B. A video support of the robot test setup is also available online at: https://www.youtube.com/watch?v=zoTABoDZbpk.

### Study protocols and design

**Fig. 3:**
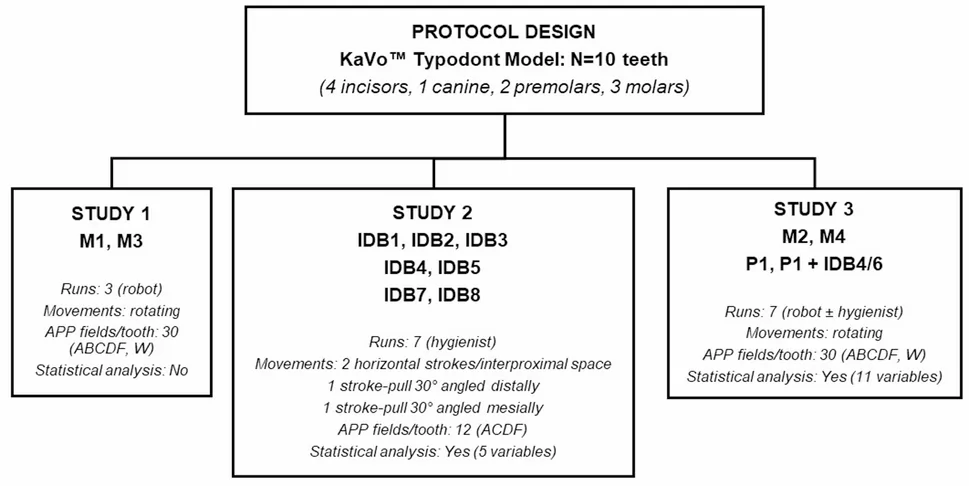
Study design and protocols

Three study protocols were used to assess and compare the cleaning efficacy of the manual toothbrushes and IDBs, as well as to evaluate the cleaning efficacy of the hybrid powered-mode toothbrush used alone and as a part of a combined regimen with different-sized IDBs. A summary of the three study protocols is provided in Fig. 3. Before each test run, a new toothbrush was mounted onto the robot testing device and the typodont model was covered with the simulated plaque using a highly standardized process that ensured 100% coverage of all planimetric fields. For the test runs involving the manual and hybrid powered-mode toothbrushes (Study 1 and Study 3), the robot was programmed to perform a rotating movement, for 2 min, and the brushing force was calibrated to 3.5 N. For the test runs involving the IDBs (Study 2), a hygienist performed four movements: two horizontal strokes per interproximal space, one stroke-pull angled distally at 30° and one stroke-pull angled mesially at 30°. The IDBs were washed in clean water to remove all residual simulated plaque before proceeding to the next interproximal space. The results of the IDB tests were used to select the optimal size of the IDBs to be used as part of the combined regimen with the hybrid powered-mode toothbrush. The selected IDBs and the hybrid powered-mode toothbrush were then assessed in combination, using the same IDB brushing parameters performed by the hygienist and the standard robotic brushing programme for the hybrid powered-mode toothbrush.

### Statistical analysis

For comparisons of manual toothbrush efficacy and hybrid powered-mode toothbrush efficacy, overall simulated plaque removal percentage values, as well as values for the 10 individual variables (buccal and lingual surfaces, ABCDF buccal and lingual gum-line fields, between-tooth mesial and distal surfaces, and W mesial and W distal root areas) were evaluated using descriptive statistics (mean ± standard deviation; SD) for the manual brushes evaluated in Study 1 (exploratory study, number of runs: 3) and, after Kolmogorov-Smirnov analysis of the distribution of the data, using the non-parametric Mann-Whitney *U* test for the manual and hybrid powered-mode brushes and combined regimen evaluated in Study 3 (number of runs: 7). For evaluation of the IDBs (Study 2), the distribution of the simulated plaque removal percentage values for the four individual variables (mesial and distal interdental surfaces, and buccal and lingual ACDF fields), as well as overall simulated plaque removal percentage values, were first analysed using the Kolmogorov-Smirnov test. Data for IDBs grouped according to size (small, medium or extra-large) were then analysed and compared using the Levene test followed by the independent two-sample *t* test, or the non-parametric Mann-Whitney *U* test, as appropriate.

Statistical analyses were performed using the IBM SPSS Professional Statistics 28, R 4.1.2 (Study 2), and IBM SPSS Statistics Premium (release 25, 64-bitversion; Study 3) software packages. Outlying data, defined as simulated plaque removal percentage values outside the 3* interquartile range of the variable distribution, were excluded from the analysis. Significance levels were set at α = 0.10 for the Kolmogorov-Smirnov and Levene tests, and at α = 0.05 (5%, *p* ≤ 0.05) for the *t* test and Mann-Whitney *U* test.

## Results

### Evaluation of manual toothbrush efficacy

**Fig. 4:**
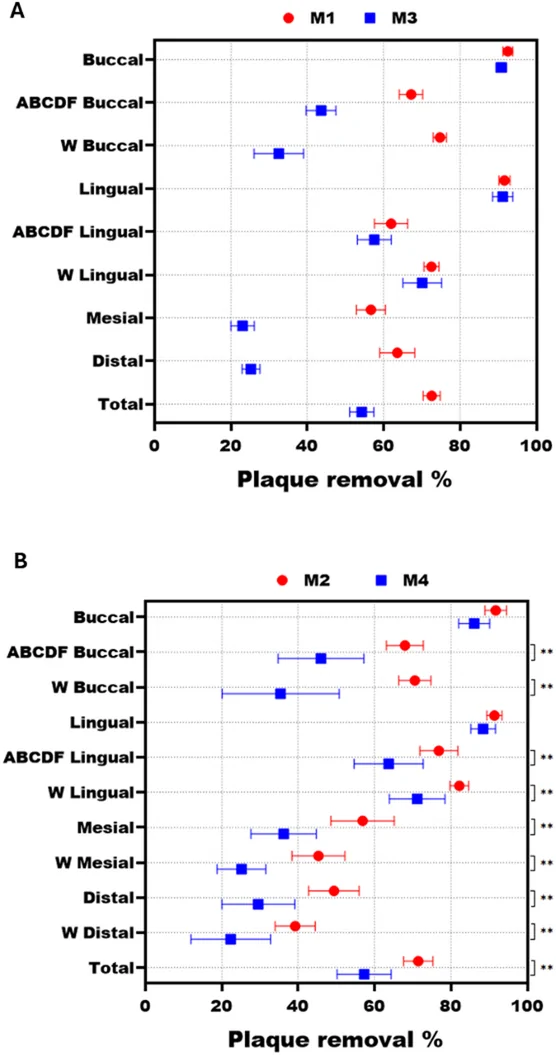
Comparison of the percentage simulated plaque removal efficacy of manual toothbrushes overall and across individual sites. Data points represent mean values and error bars the standard deviation. **A**. M1 vs. M3, number of replicated runs: *n* = 3. **B**. M2 vs. M4, number of replicated runs, *n* = 7. ** *p* ≤ 0.01 for between-brush comparisons according to the Mann-Whitney *U* test

The results of comparisons between the manual toothbrushes evaluated in Study 1 (M1 vs. M3) and Study 3 (M2 vs. M4) are shown Fig. 4, and in Additional Table 1. The M1 toothbrush (3024, soft, 11-mm, conical-shaped bristles), showed the highest overall level of cleaning efficacy, with a mean percentage simulated plaque removal value across all sites of 72.6 ± 2.2%, whereas the lowest level of cleaning efficacy was observed for the M3 toothbrush (5460, extra-soft, 9-mm, rounded cylindrical bristles) with an overall mean percentage simulated plaque removal value of 54.3 ± 3.2% (Fig. 4A and in Additional Table 1A). The M1 and M3 brushes showed similar levels of simulated plaque removal efficacy on smooth surfaces (> 90% for both buccal sites and lingual sites). However, higher levels of efficacy for M1 were observed for hidden accumulation sites, such as the mesial (M1: 56.6 ± 3.8% vs. M3: 23.1 ± 3.0%) and distal fields (M1: 63.6 ± 4.6% vs. M3: 25.3 ± 2.3%), as well as for buccal at-risk fields next to the gum line (buccal ABCDF; M1: 67.2 ± 3.1% vs. M3: 43.6 ± 3.9%) and the buccal root field (W buccal; M1: 74.7 ± 1.7% vs. M3: 32.5 ± 6.5%).

The M2 toothbrush (4176, soft, 11-mm, rounded cylindrical bristles) showed the second highest level of cleaning efficacy overall, and was significantly more effective than the M4 toothbrush (1560, soft, 9-mm, rounded cylindrical bristles): 71.4 ± 3.8% vs. 57.3 ± 7.1% respectively (*p* < 0.01) (Fig. 4B and in Additional Table 1B and C). As with M1 and M3, similar levels of simulated plaque removal efficacy were observed for the two brushes on smooth surfaces (buccal and lingual; > 86%, *p* > 0.05) and the difference in the overall cleaning efficacy of the toothbrushes was driven by more efficient simulated plaque removal for the M2 toothbrush at hidden accumulation sites (mesial and distal fields, 56.9 ± 8.3% and 49.4 ± 6.6% respectively), buccal at-risk fields (buccal ABCDF 67.9 ± 4.8%) and buccal root fields (W buccal 70.6 ± 4.2%) with *p* < 0.01 for between-brush differences for all fields. In addition, the M2 toothbrush showed higher levels of efficacy for the lingual gum-line fields (lingual ABCDF 76.8 ± 4.9%, *p* = 0.004) and the lingual root field (W lingual 82.2 ± 2.5%, *p* = 0.003).

### Evaluation of interdental brush efficacy

**Fig. 5:**
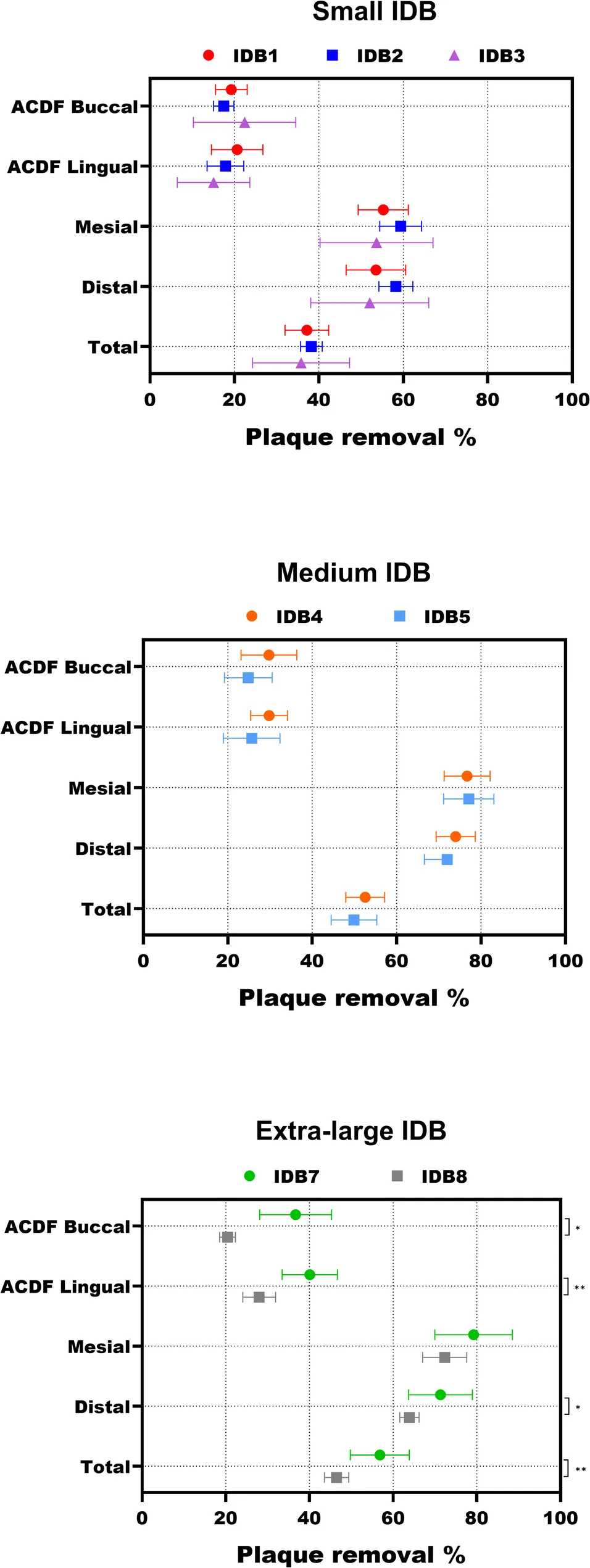
Comparison of the percentage simulated plaque removal efficacy of interdental brushes (IDBs). IDBs were compared overall and across individual between-tooth (mesial and distal) surfaces and interproximal at-risk sites (ACDF) according to brush size: **A**. small (IDB1, IDB2 and IDB3**) B**. medium (IDB4 and IDB5), and **C**. extra-large (IDB7 and IDB8). Data points represent mean values and error bars the standard deviation. Number of replicated runs: *n* = 7 for all tests, except for IDB8 at the ACDF buccal site (*n* = 6). *p ≤ 0.05 and ** *p* ≤ 0.01 for between-brush comparisons according to either the *t* test (mesial, distal and total values) or the Mann-Whitney *U* test (buccal and lingual ACDF field values)

The results of comparisons between IDBs grouped according to size (small: 0.8 mm, medium: 1.3–1.5 mm, and extra-large 2–2.2 mm) are shown in Fig. 5 and in Additional Table 2. Compared to the medium and extra-large IDBs, the small IDBs (IDB1, IDB2, and IDB3) were the least well-adapted IDBs for use on the tooth model, with mean percentage values for overall cleaning efficacy ranging from 35.8 ± 11.5% to 38.2 ± 2.6%. No significant differences were observed for cleaning efficacy between the different small brush models at any of the sites (Fig. 5A and Additional Table 2 A and D; *p* > 0.05). The small brushes showed the highest levels of cleaning efficacy for the mesial and distal sites (means of 52.1 ± 14.0% to 59.4 ± 5.0%) and the lowest levels of cleaning efficacy for the at-risk interproximal buccal and lingual fields (ACDF means: 15.1 ± 8.6% to 22.4 ± 12.1%).

The medium IDBs (IDB4 and IDB5) generally seemed better adapted for use on the tooth model than the small brushes, with overall mean percentage values of 52.6 ± 4.6% and 49.9 ± 5.4% for IDB4 and IDB5, respectively (Fig. 5B and Additional Table 2B and D). Notably, mean levels of cleaning efficacy for the medium IDBs tended to be higher than those for small IDBs at the poorly accessible mesial and distal sites (72.0 ± 5.4% to 77.1 ± 6.0% vs. 52.1 ± 14.0% to 59.4 ± 4.9% for the medium and small IDBs, respectively; *p* < 0.001 for the medium IDBs vs. small IDBs 1 and 2), and, in the case of IDB4, for the at-risk interproximal buccal and lingual ACDF fields (*p* < 0.01), with mean values for the medium IDBs ranging from 24.9 ± 5.7% to 29.8 ± 4.4%. No statistically significant differences were observed for mean cleaning efficacy between the two medium IDBs (IDB4 and IDB5) at any of the sites (*p* > 0.05; Fig. 5B and Additional Table 2B and D).

The overall mean percentage cleaning efficacy values for the extra-large IDBs were 56.8 ± 7.1% and 46.5 ± 2.9% for IDB7 and IDB8, respectively, and were comparable to those of the medium IDBs (*p* > 0.05 for all comparisons of total efficacy; Fig. 5C and Additional Table 2 C and D). However, despite the similarity in size, mean percentage simulated plaque removal values for IDB7 were significantly higher than those for IDB8, overall (*p* < 0.01) and at the distal site (*p* < 0.05), and the buccal and lingual ACDF at-risk interproximal areas (*p* < 0.05 and *p* < 0.01, respectively).

### Evaluation of hybrid powered-mode toothbrush efficacy, with or without IDBs

**Fig. 6:**
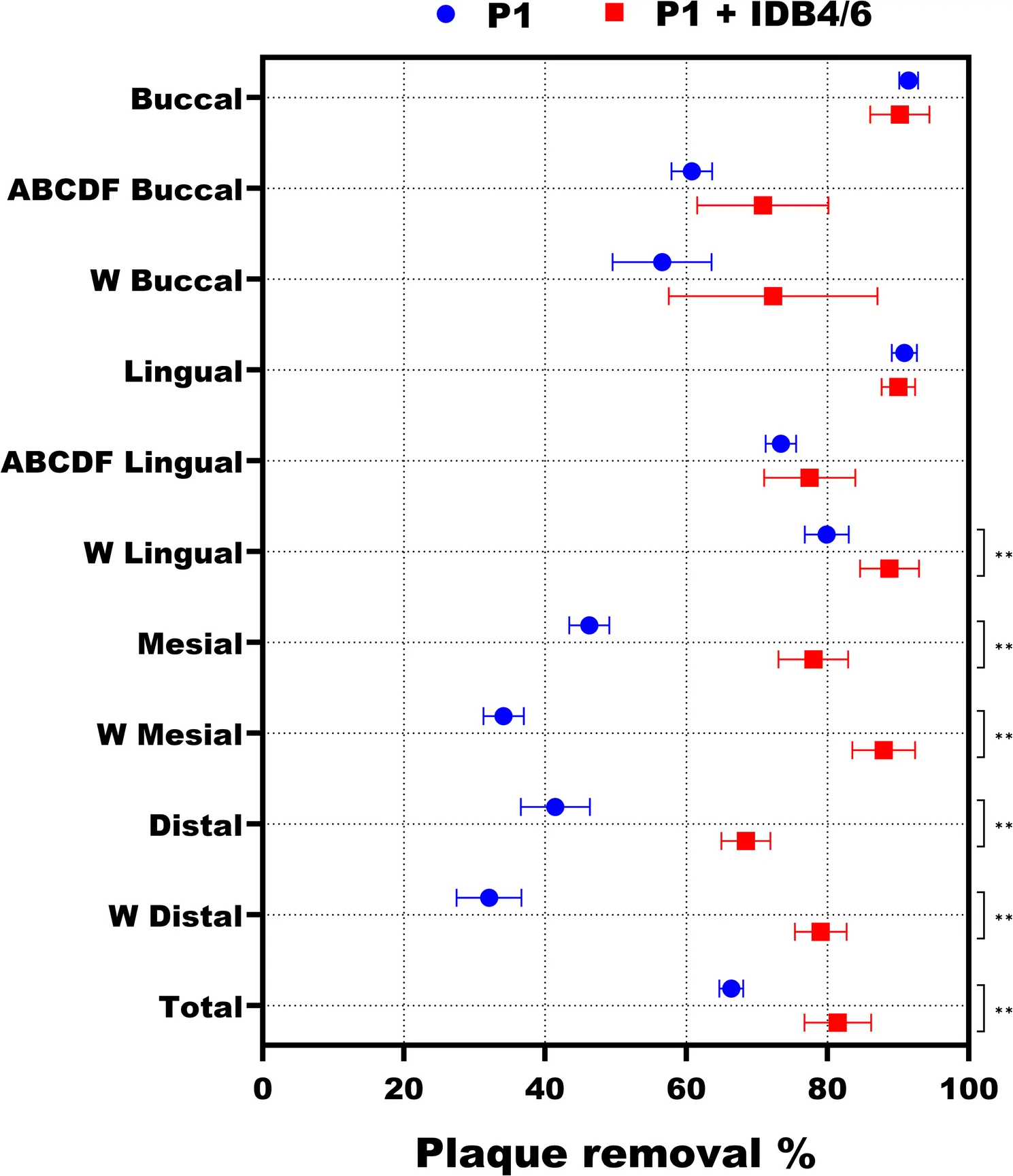
Comparison of the percentage simulated plaque removal efficacy of the hybrid powered-mode toothbrush (P1) with/without interdental brushing. Hybrid powered-mode toothbrush (P1) efficacy was compared with or without the use of IDB4 (medium IDB) and IDB6 (large IDB), overall and across individual surfaces (buccal, lingual and between teeth: mesial and distal) and at-risk gum-line fields (ABCDF, W mesial, and W distal). Data points represent mean values and error bars the standard deviation. Number of replicated runs: *n* = 7 for all tests. ** *p* ≤ 0.01 for between-regimen comparisons according to the Mann-Whitney *U* test

When evaluated alone, the hybrid powered-mode toothbrush (P1) achieved an overall mean level of cleaning efficacy of 66.4 ± 1.7% and showed excellent levels of simulated plaque removal from the smooth buccal and lingual areas (> 90%; Fig. 6 and Additional Table 3 A), similar to the levels observed for the best-performing manual toothbrushes (M1 and M2; Fig. 4 and Additional Table 1A and B).

Statistically significant higher levels of cleaning efficacy were achieved by combined brushing with the hybrid P1 toothbrush and interdental brushing with IDB4 (the most efficient medium IDB) and a large IDB, IDB6 (Fig. 6 and Additional Table 3 A and B). The overall mean percentage simulated plaque removal value for the combined brushing was 81.5 ± 4.7% (*p* < 0.01 for the difference between the two regimens). Notably, when compared with use of P1 alone, the combined regimen led to statistically significant (*p* < 0.01) improvements in simulated plaque removal from the hard-to-reach distal (P1: 41.4 ± 4.9% vs. P1 + IDB4/6: 68.4 ± 3.5%) and mesial (P1: 46.2 ± 2.8 vs. P1 + IDB4/6: 78.0 ± 4.9) interdental surfaces, and the W mesial (P1: 34.1 ± 2.9% vs. P1 + IDB4/6: 88.0 ± 4.4%) and W distal (P1: 32.1 ± 4.6% vs. P1 + IDB4/6: 79.0 ± 3.7%) root fields.

## Discussion

This series of studies assessing the cleaning efficacy of several commercially available manual toothbrushes and IDBs, and of a hybrid powered-mode brush with or without interdental brushing, further demonstrated the feasibility and adaptability of in vitro testing, and in particular, the robot simulation–APP system for conducting standardized and comparative evaluations of mechanical simulated plaque removal. The present study was not designed to distinguish the relative contribution of each individual design parameter of the tested dental materials. Our analysis of four manual toothbrushes allowed for objective evaluations of the potential impact of bristle design on cleaning efficacy and provided valuable insights for the future development of more efficient manual toothbrush models. Our evaluation of a range of different-sized IDBs was conducted using a standardized protocol performed by a hygienist. Our findings highlighted the importance of choosing appropriately sized IDBs to optimize brushing effectiveness in the typodont model. Finally, we demonstrated the potential benefits for optimizing plaque removal by combining robot programme using hybrid toothbrush in powered mode with hygienist-based IDB protocol.

The four manual toothbrushes tested in our study differed in terms of bristle number, hardness, height, and shape. Our findings showed that these variations in design had little impact on the ability of the brushes to effectively remove simulated plaque from the smooth and easily accessible tooth surfaces under test conditions. The higher levels of overall efficacy observed for the more performant brushes (M1 and M2) were driven by improved simulated plaque removal from hidden or more difficult to reach plaque accumulation sites in the typodont model (interdental surfaces, gum-line areas, and root fields). The comparison between M1 and M3 indicated that bristle hardness and shape may have had an impact on cleaning efficacy under test conditions, with the soft, and conical-shaped bristles of the M1 brush resulting in higher levels of simulated plaque removal from all at-risk surfaces and areas, and particularly on the slightly more accessible buccal gum line and root field areas, than the extra-soft and rounded cylindrical bristles of the M3 brush. These analyses also suggested that greater tuft height may be beneficial for accessing harder to reach interdental, gum-line and root areas. These findings support those of Axe et al. (2024), in which use of the robot simulation–APP system in the rotating cleaning mode (simulating the Modified Bass Method [25]), as used in the current study, also identified greater tuft height and a soft brush as being more efficient for simulated plaque removal under test conditions [15]. In addition, our comparison between M2 and M4 indicated that a greater number of bristles per brush improved cleaning efficacy of the typodont model. However, further studies are required to determine the optimal number and configuration of bristles, as overall, the poorest performing brush (M3) contained the highest number of bristles. It should be noted that relatively high and consistent pressure was applied during the robot brushing programme (3.5 N). It might have influenced outcomes with different stiffness and filament lengths, and it also deviated from reported in-use adaptive behavior by consumers. In this context, it may be of value to test the impact of slighter pressure on simulated plaque removal efficacy by the different toothbrushes, but also to measure the toothbrush pressure applied by subjects in real life conditions of use in future studies.

Our analysis of the different-sized IDBs highlighted the importance of adapting the diameter of the brush to the anatomical characteristics of the model teeth and the surrounding model gum level to optimize plaque removal. Small IDBs were insufficient to allow for adequate cleaning of the interproximal regions and interdental spaces of the typodont model, with overall percentage efficacy values below 40%. Improved cleaning efficacy was observed with the medium IDBs and extra-large IDBs. However, the observed similarity in cleaning efficacy between medium and extra-large brushes showed that brush diameters ≥ 2 mm resulted in only limited or no improvements in simulated plaque removal efficacy under test conditions, indicating that the extra-large brushes were too large for efficient cleaning of the areas tested. These findings are consistent with the conclusions drawn by several authors based on evidence from clinical studies, which identified the need for individual assessments and calibration of IDBs according to the characteristics of each tooth, gingival area, and size of the interdental space in order to optimize disruption of oral biofilms in interproximal spaces and significantly improve periodontal outcomes such as bleeding and inflammation [5, 8, 22, 26–28]. Apart from size, other design characteristics (e.g., brush and bristle shape) seemed to have little impact on cleaning efficacy in the current study, with no significant differences in cleaning efficacy observed between the different designs of small and medium IDBs. However, IDB7 was found to be more efficient at simulated plaque removal than IDB8, with this difference potentially related to differences in brush shape or bristle design. Further studies are required to more accurately assess the impact of these design characteristics on interproximal and interdental plaque removal.

Numerous clinical studies and subsequent systematic reviews and meta-analyses have provided evidence supporting the oral health benefits of combining brushing with manual or powered toothbrushes and the use of interdental cleaning devices, leading to inclusion of this dual approach in recommendations from professional dental associations and consensus guidelines [1, 6, 7, 13, 23, 29]. To our knowledge, it is the first time that this combined approach is used in a standardized in vitro testing system. The brush used in the current study was a hybrid model in powered (sonic) mode with previously demonstrated clinical efficacy on dental plaque reduction. After only 7 days of twice daily use, it proved to be significantly better than an American Dental Association (ADA) standard flat-trim manual toothbrush and this difference was even more evident after 14 days [20]. After a single use, it also significantly reduced dental plaque and was as effective as an oscillating-rotating powered brush in plaque removal [21]. In the current study, when tested alone in the powered-mode using the in vitro system, this hybrid powered-mode brush was found to be similarly effective as the best-performing manual toothbrushes for overall simulated plaque removal. The choice of IDBs used in the combined regimen was based on the results of the IDBs evaluation, which suggested that a combination of the best-performing medium IDB with a large IDB would result in maximal cleaning efficiency. Our results showed that the combined use of the hybrid powered-mode brush with the appropriately sized IDBs led to a significant improvement in cleaning efficiency and achieved a high level of overall percentage simulated plaque removal under the test conditions. The improved efficacy of the combined test routine was mainly driven by improved simulated plaque removal from interdental surface and root field areas. These findings therefore highlight the potential benefits of the combined routine for improving cleaning of at-risk accumulation sites and show that the robot simulation–APP testing system could potentially be used in early product development to assess the impact of new design features of hybrid and powered brushes, and, when combined with the hygienist protocol, could allow for more rapid, objective and cost-effective pre-clinical evaluations of new devices used as part of a brushing routine.

Use of the innovative in vitro test system, including the clinically validated 6-axis robot toothbrushing simulation coupled with APP to evaluate simulated plaque removal, was one of the main strengths of the study. Indeed, the robotic brushing programme used here (standardized brush angulation and rotating movement, 3.5 N brushing force and 2-min brushing time) was identical to that shown by Lang et al. (2014) [24] to produce clinically relevant cleaning patterns that mimicked toothbrushing by pre-trained human subjects according to a standardized routine (e.g., highly trained undergraduate dental students, carrying out supervised bushing movements with a calibrated force after a 3-day plaque regrowth interval following dental cleaning). In addition, APP allowed for rapid and reproducible evaluation of the cleaning efficacy of the toothbrushes across 30 distinct tooth fields, allowing the impact of design features to be assessed on the removal of simulated plaque not only from smooth tooth surfaces, but also from key at-risk accumulation sites, such as interdental spaces, and gum-line and root areas, under test conditions. The same methodology, but without assessment of the at-risk root fields (W buccal and W lingual and W mesial and W distal), was used previously by Axe et al. (2024) [15] to identify the design features of a different range of manual toothbrushes that potentially impacted cleaning efficacy. A key new element in the current study was the introduction of the standardized hygienist-based protocol coupled with APP detection across 12 interdental and interproximal fields to allow objective assessment of cleaning efficacy of the IDBs, alone and then in combination with robot-simulated powered brushing to demonstrate the benefits of a simulated oral hygiene routine. However, our study has several limitations. The first in vitro study was conducted as an exploratory study to verify the parameters and limits of detection the test system and, as only 3 test runs were performed for each brush, the data obtained could only be described using descriptive statistics. Moreover, only rotating brushing technique and flat-trim brush design were used in the robot programme. Further analyses using alternative brushing techniques (e.g., commonly used vertical or horizonal movements [15]) and different trim designs may be of value in future studies. In addition, as noted by Lang et al. (2014) [24], the brushing force (3.5 N) and total brushing time (2 min) used in the robot programme were above the mean values reported in a clinical study evaluating the toothbrushing habits of uninstructed adults (3.2 N and 96 s, respectively) [11], but were still considered within the acceptable range to provide relevant information for comparative in vitro assessments of device cleaning efficacy. Moreover, an earlier clinical study indicated that increased brushing force up to a threshold of around 4.0 N (above the force used in the current study) was associated with increased brushing efficacy [30]. It should also be noted that, as the clinical validation of the robot programme was conducted using a best-case scenario (highly standardized brushing regime carried out by trained individuals) [24], our in vitro findings only provide potential guidance for early product development, and clinical testing remains essential for further evaluations of user acceptability and overall impact on cleaning efficacy. Finally, it could also be interesting to develop new disease-mimicking typodont models that could be used to assess simulated dental plaque removal in cases where gums and teeth are damaged (e.g., periodontal disease) and therefore allow in vitro testing of toothbrush efficacy in simulated pathologic conditions.

## Conclusions

Our data indicate that in vitro testing could be a valuable tool for identifying potential design characteristics that may improve the cleaning efficacy of toothbrushes, potentially allowing for more rapid assessment and development of future innovations in the field. Evaluation of IDBs using the typodont model and APP system indicated the importance of individual assessments of tooth and periodontal characteristics to ensure the use of appropriately sized IDBs to optimize the potential cleaning efficacy of these devices. Finally, our testing strategy combining the robot system with a standardized hygienist protocol could be useful for evaluating the potential benefits of combined toothbrushing and interdental cleaning routines, and may provide guidance for the development and clinical testing of combinations of new products and device ranges.

## Supplementary Information

Supplementary Material 1. Additional Table 1: Simulated plaque removal efficacy of the manual toothbrushes. Percentage plaque removal for A. M1 and M3 and B. M2 and M4. C. p values for between-brush comparisons (M2 vs M4) according to the Mann-Whitney U test.

Supplementary Material 2. Additional Table 2: Simulated plaque removal efficacy of the interdental brushes according to size. Percentage plaque removal for the A. small, B. medium and C. large interdental brushes. D. p values for between-brush comparisons according to the t test and the Mann-Whitney U test.

Supplementary Material 3. Additional Table 3: Simulated plaque removal efficacy of the hybrid powered-mode toothbrush (P1), with and without use of a medium (IDB4) and large (IDB6) interdental brushes A. Percentage plaque removal and B. p values for between-regimen comparisons according to the Mann-Whitney U test.

## Data Availability

The datasets used and/or analysed during the current study are available from the corresponding author on reasonable request.

## Abbreviations

APP: Automatic Plaque Planimetry
ADA: American Dental Association
IDB: Interdental Brush
IDB1, IDB2, IDB3, IDB4, IDB5, IDB6, IDB7, IDB8: Different sizes and models of interdental brushes tested
M1, M2, M3, M4: Manual toothbrush models tested
P1: Hybrid powered-mode toothbrush model tested
SD: Standard deviation
W: Root field zones (W mesial, W distal, W buccal, W lingual)
